# Novel full‐spectral flow cytometry with multiple spectrally‐adjacent fluorescent proteins and fluorochromes and visualization of in vivo cellular movement

**DOI:** 10.1002/cyto.a.22725

**Published:** 2015-07-28

**Authors:** Koji Futamura, Masashi Sekino, Akihiro Hata, Ryoyo Ikebuchi, Yasutaka Nakanishi, Gyohei Egawa, Kenji Kabashima, Takeshi Watanabe, Motohiro Furuki, Michio Tomura

**Affiliations:** ^1^ FCM Business DepartmentLife Science Business DivisionMedical Business UnitSony CorporationMinato‐KuTokyo108‐0075Japan; ^2^ Concept Development DepartmentApplication Technology Development DivisionSystem R&D Group, RDS Platform, Sony CorporationShinagawa‐KuTokyo141‐0001Japan; ^3^ Center for Innovation in Immunoregulative Technology and Therapeutics, Kyoto University Graduate School of MedicineYoshida‐KonoeKyoto606‐8501Japan; ^4^ Department of DermatologyKyoto University Graduate School of MedicineKyoto606‐8501Japan; ^5^ Laboratory of ImmunologyFaculty of Pharmacy, Osaka Ohtani University3‐11‐1 NishikiorikitaTondabayashi‐CityOsaka Prefecture584‐8540Japan; ^6^ The Tazuke‐Kofukai Medical Research Institute/Kitano Hospital2‐4‐20 OhgimachiKita‐KuOsaka530‐8480Japan

**Keywords:** spectral flow cytometry, separation of spectrally‐adjacent fluoroprobes, photoconvertible fluorescent protein, immune cell movement

## Abstract

Flow cytometric analysis with multicolor fluoroprobes is an essential method for detecting biological signatures of cells. Here, we present a new full‐spectral flow cytometer (spectral‐FCM). Unlike conventional flow cytometer, this spectral‐FCM acquires the emitted fluorescence for all probes across the full‐spectrum from each cell with 32 channels sequential PMT unit after dispersion with prism, and extracts the signals of each fluoroprobe based on the spectral shape of each fluoroprobe using unique algorithm in high speed, high sensitive, accurate, automatic and real‐time. The spectral‐FCM detects the continuous changes in emission spectra from green to red of the photoconvertible protein, KikGR with high‐spectral resolution and separates spectrally‐adjacent fluoroprobes, such as FITC (Emission peak (Em) 519 nm) and EGFP (Em 507 nm). Moreover, the spectral‐FCM can measure and subtract autofluorescence of each cell providing increased signal‐to‐noise ratios and improved resolution of dim samples, which leads to a transformative technology for investigation of single cell state and function. These advances make it possible to perform 11‐color fluorescence analysis to visualize movement of multilinage immune cells by using KikGR‐expressing mice. Thus, the novel spectral flow cytometry improves the combinational use of spectrally‐adjacent various FPs and multicolor fluorochromes in metabolically active cell for the investigation of not only the immune system but also other research and clinical fields of use. © 2015 The Authors. Cytometry Part A Published by Wiley Periodicals, Inc. on behalf of ISAC

## Introduction

Flow cytometry has become a useful methodology for high‐speed and sensitive analysis of fluorescence signals for many research fields, including molecular biology, medicine, immunology, pathology, plant biology, and marine biology 1, 2, 3. Mutual development of both hardware and reagents enabled us to achieve a multiparameter analysis of individual cells. Instrument manufacturers have developed multiple laser (355 nm, 405 nm, 488 nm, 561 nm, etc.) flow cytometers (FCM) with independent resulting signal detection paths. Array of brighter and spectrally diverse fluorochromes, such as the Brilliant Violet^™^ dyes for violet lasers 4 have been developed. In parallel, various kinds of fluorescent proteins **(**FPs) from blue to far‐red have been developed 5, 6, 7, 8, 9, 10, 11, 12, 13 and FP‐tagged proteins are expressed in cell lines and in animals. Additionally, fluorescence resonance energy transfer (FRET) constructs of fusion proteins consisting of donor and acceptor FPs are used for elucidating protein‐protein intermolecular interactions, cell signaling, cell death, and other functional cellular states 9, 14, 15, 16, 17. Thus, the genetically modified expression of FPs has become a general approach for both cell‐type specific labeling and functional monitoring of protein production and signaling.

So far FCM and fluorochromes have been developed mainly for investigation of immunology. Binding fluorochrome‐conjugated antibodies to cells for flow cytometric analysis is a common and essential method to analyze phenotypes and characteristics of single cells in mixed populations. Therefore, the demand for simultaneous detection of spectrally‐adjacent FPs and multiple fluorochromes in biological research has been increasing. We have established a novel system for visualizing cellular movement in vivo using photoconvertible FPs in Kaede‐transgenic mice 18 and KikGR‐expressing mice 19, 20. This technique enables us to label cells with photoconvertible FPs which will change their fluorescence emission from green to red with exposure to violet light and allows us to monitor cellular movement between lymphoid organs and peripheral tissues with FCM. Our approach revealed the novel concepts of T cell recirculation in maintenance of their own function 21 and the critical role of skin‐migratory regulatory T cells in the termination of contact hypersensitivity response 22. Thus, photoconvertible FPs are an effective tool to investigate the immune system based on spatiotemporal regulation of immune cells in the entire body. Information of cellular movement in multilineages of immune cells, such as T cells, B cells, dendritic cells, and their subpopulations in the same samples is highly informative and is critical for making whole images comparing immune cell dynamics in the steady state and pathophysiological condition. However, because both non‐photoconverted green and photoconverted red Kaede and KikGR in the mice have strong fluorescence intensity and broad emission spectra, only three to five additional fluorochromes can be used simultaneously with the photoconvertible FPs by conventional nonspectral FCM. The use of multi‐FP, multilineage analysis has been limited. Spectral flow cytometer (spectral‐FCM) enables simultaneous detection of multiple FPs with conventional fluorochromes.

While conventional FCM uses dichroic mirrors and band‐pass filters to segment and filter emitted fluorescence light, spectral flow cytometry is a new technology for detecting fluorescent signals derived from individual cells. Spectral‐FCM disperses emission fluorescence from individual cells with prisms or diffraction grating, acquires the full fluorescence spectra with photomultiplier (PMT) or charge coupled device (CCD), and unmixes component signals from each fluoroprobe 23, 24, 25, 26, 27, 28, 29. A spectral confocal laser scanning microscopy with similar optical schemes of the spectral‐FCM 30 have been separated FPs and fluorochromes 31, displayed each fluorescence spectrum from cells, and discriminated autofluorescence 32. Therefore, a spectral‐FCM is expected to become a powerful tool utilizing advanced spectral analysis features. Although there are several reports including interesting technologies, design ideas, and analysis algorithms of spectral‐FCM, the development of practical biological applications has not been previously shown because of insufficient sensitivity and data acquisition speed.

Here we present a novel spectral‐FCM (already commercialized as SP6800). The spectral‐FCM with a unique optical configuration and algorithm provides high speed, high sensitive, accurate, automatic analysis, and real‐time spectral unmixing from all fluorescent signals even in a multicolor analysis of spectrally‐adjacent FPs and fluorochromes. Moreover, the spectral‐FCM can measure and subtract autofluorescence of each cell providing increased signal‐to‐noise ratios and improved resolution of dim samples. These advances make it possible to perform multilineage analysis of immune cell movement in the entire body. This novel spectral‐FCM improves the combinational use of spectrally‐adjacent various FPs (including photoconvertible FPs) and multicolor fluorochromes for the investigation of not only the immune system but also other research and clinical fields of use.

## Materials and Methods

### Design and Key Components of the Spectral‐FCM

**Figure 1:**
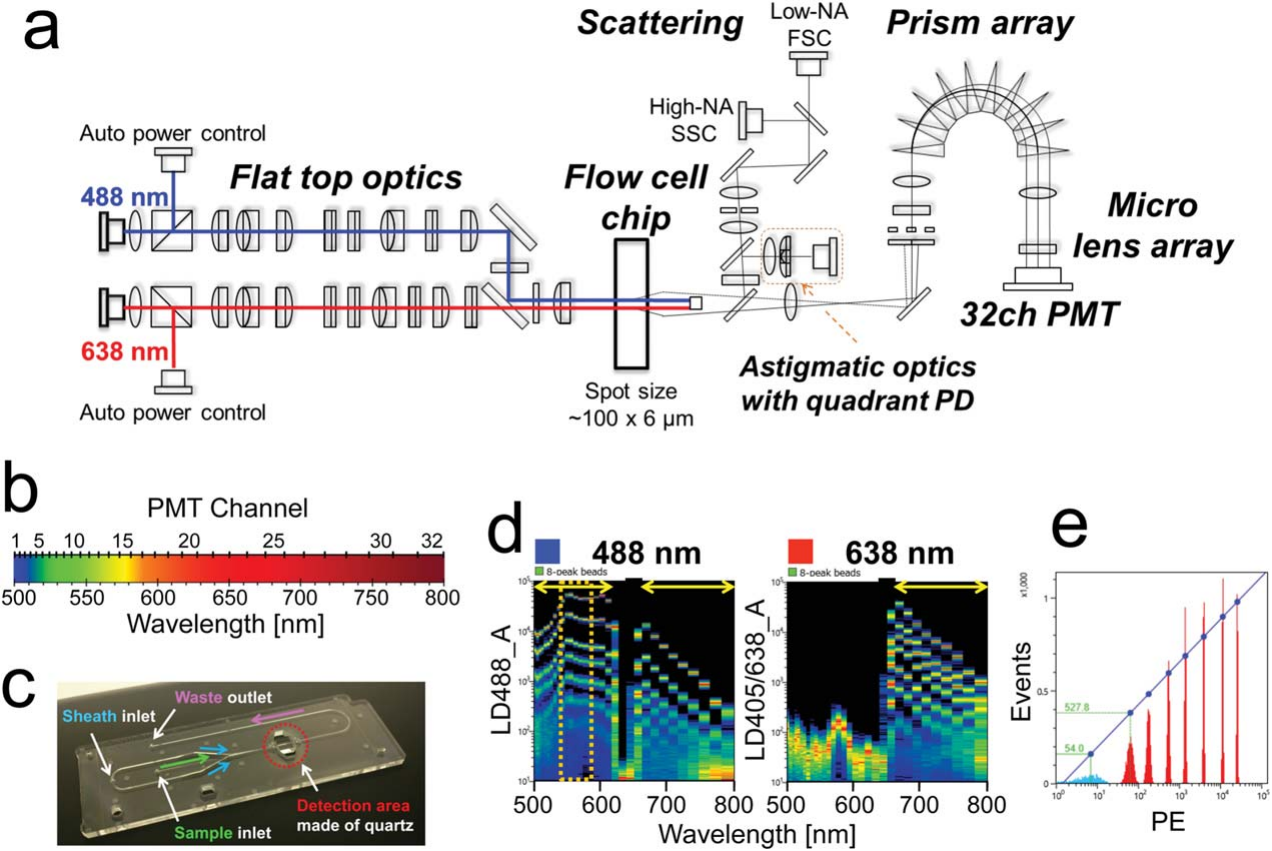
Design and characterization of the spectral‐FCM. **a**) Unique optical designs adopted by the spectral FCM, 488/638 nm non‐coaxial dual lasers, flow cell chip, amorphous‐Si PD, quadrant PD, prisms, micro lens array, and 32ch PMT. **b**) Wavelength ranges of individual each channel in 32ch PMT. More channels are applied in the blue‐green region than red one in order to increase the wavelength resolution in that region. This optical configuration was achieved by using unique prism optics. **c**) Image of a flow cell chip. Blue, green, and pink arrows mark sheath, sample, and waste flows, respectively. Red dash circle marks detection area made of quartz. **d**) Spectrum charts acquired by the detection of Ultra Rainbow 8‐peaks beads. Acquired spectra by 488 nm laser excitation (left) and 638 nm (right) are shown, respectively. Wavelengths from 500 nm to 800 nm as the abscissa and fluorescent intensities as the ordinate are indicated. The colors in charts indicate densities of each channel. Red shows high density, green shows middle, and blue shows low, respectively. The 20^th^−23^rd^ channels in the 32ch PMT are shielded by a mask to prevent 638 nm laser shining into the PMT and not detected in the dual laser mode (488 nm and 638 nm excitations). Yellow arrows in plots show regions where fluorescence data are used when the spectral‐FCM is run in dual laser mode. **e**) Histogram of acquired data by the detection of Ultra Rainbow 8‐peaks beads in PE region (Fig. 1d; left, orange dash line). MESF and linearity was calculated depending on this data. 54.0 and 527.8 shows mean fluorescent intensities from the lowest intensity beads (light blue) and the second lowest intensity beads (red, right next to light blue), respectively.

The optical schematic diagram of the spectral‐FCM is shown in Figure 1a. The SP6800 is equipped with 488/638 nm lasers, flow cell chip, photodiode (PD), quadrant PD, 10 consecutive prisms, micro lens array, and 32‐channel linear array PMT (32ch PMT). The spectral‐FCM offers a unique detection of fluorescent light capturing all the emitted sample fluorescence as spectra ranging from 500 nm to 800 nm.

The excitation laser beam power profile is formed into flat top curve by focusing lenses in a direction perpendicular to the flow direction with 40 µm width at 80% of the peak intensity, where the beam profile of flow direction is Gaussian and 6 µm at 1/*e* ^2^. 488/638 nm laser spots are spatially separated and laser powers at the surface of the flow cell chip are 40 mW for 488 nm and 60 mW for 638 nm, respectively. Each laser power is constantly monitored and controlled automatically. The flat top beam shape always provides quantitative fluorescence intensity even if the stream line of each cell changes in the microfluidic channel because the excitation power density is nearly constant.

Ten consecutive prisms are highly transparent and coated with original anti‐reflecting films. A custom micro lens array assembly then focuses each band of light onto a specific channel of the PMT array, which avoids losing fluorescent photons to a boundary mask. The 32ch PMT detects light in the visible spectrum from 500 nm to 800 nm. In order to achieve high‐resolution of wavelength in the blue‐green region where a lot of FPs and fluorochromes emit, the optical system has been designed so that more channels are assigned for the blue‐green region (Fig. 1b). When the spectral‐FCM is running in the dual laser mode (488 nm and 638 nm excitations), 20th to 23rd channels (617–662 nm) in the 32ch PMT are automatically shielded to prevent 638 nm laser shining into the PMT by inserting a mask. For each single cell, acquired fluorescent data of 28 channels (1st–19th and 24th–32nd channels) by 488 nm excitation and nine channels (24th–32nd channels) by 638 nm excitation, totally 37 channels of fluorescence data are used for further processing (Fig. 1d). On the other hand, when running in the single laser mode (488 nm excitation), fluorescent data of 32 channels are acquired and used for further processing. This spectral data collection allows for easy separation of adjacent fluorescence spectra and multicolor analysis at conventional FCM processing rates. The gain of the 32ch PMT can be controlled individually for each channel as well as the overall voltage of the entire 32ch array. The scattered light collected through the original objective lens is divided by a beam splitter and further divided by an original zonal mirror, which means the lower numerical aperture (NA) component is reflected as a forward scattering (FSC) signal, and higher NA component transmitted as a side scattering (SSC) signal. FSC and SSC are detected by amorphous‐Si PD and cell size is detected from 0.5 µm to 40 µm using the FSC signal. The first reflected scattering signal is used to control the *Z* focus position, utilizing an astigmatic focusing algorithm, as well as to control the center position of the fluidic channel utilizing a tracking algorithm with quadrant PD (Fig. 1a, orange dash line). This technology is often used for optical disk servo system, such as CDs and DVDs. As a result, the position of cells in a flow cell chip can be detected in *X* and *Y* dimensions from center in the core sample stream.

The spectral‐FCM has adopted a replaceable flow cell chip, as opposed to a conventional quartz cuvette, which is comprised of plastic plates and quartz optical detection component, in order to reduce instrument downtime when exchanging the flow cell chip (Fig. 1c). The size of the hybrid flow cell chip is 75 mm × 25 mm × 2 mm, containing microfluidic channels with several patterned channels on the base surface. There are two types of inlets, one is for the test sample and the other for sheath liquid, that latter of which splits two ways to hydrodynamically focus the sample fluid into a core stream in the central portion of the microfluidic channel. Even though the thickness of the flow cell chip is only 2 mm, the sample core is designed to be confined into the center of the detection area by both sides of the sheath flow using three dimensional hydrodynamic focusing. The typical core sample diameter is approximately 10 µm, and the size is controllable by changing sample pressure and sheath pressure independently. The optical detection area is made of quartz to minimize autofluorescence and obviate deterioration by 488 nm and 638 nm LD excitations. The advantage gained by using the hybrid flow cell chip is that the surface of detection area is kept clean against the adsorption of cells and proteins. The spectral‐FCM uses an automated chip alignment mechanism, which detects the intensity and coefficient of variation of SSC signal from align check calibration beads at each chip position and iteratively selects the optimal position. Automated alignment is useful to find the best position for the flow cell chip and for determining when it is necessary to exchange the flow cell chip in case of an undesirable clog or performance degradation due to residue on the surface of detection area.

Three sheath flow rates are factory programmed at low, mid, and high ranges, which are approximately 3 m/s, 5 m/s, and 10 m/s stream velocity and the sample flow rate is approximately 18 µL/min, 30 µL/min, and 60 µL/min, respectively. All data shown in this manuscript was taken at the mid flow rate. The sample event rate is 10,000 events per second (eps) as a nominal value dependent on sample concentration.

For signal processing, the pulse data resolution is 20‐bit height and 32‐bit area at 50 MHz sampling frequency. With additional signal processing the spectral‐FCM offers not only spectral information but also conventional flow cytometry data such as histogram, bivariate analysis, and real time dot display. The analyzed data can be exported in the form of FCS3.0 and 3.1 with/without spectral unmixing. These specifications of the spectral‐FCM are summarized in Supporting Information Table 1.

### Spectral Unmixing Algorithms

**Figure 2:**
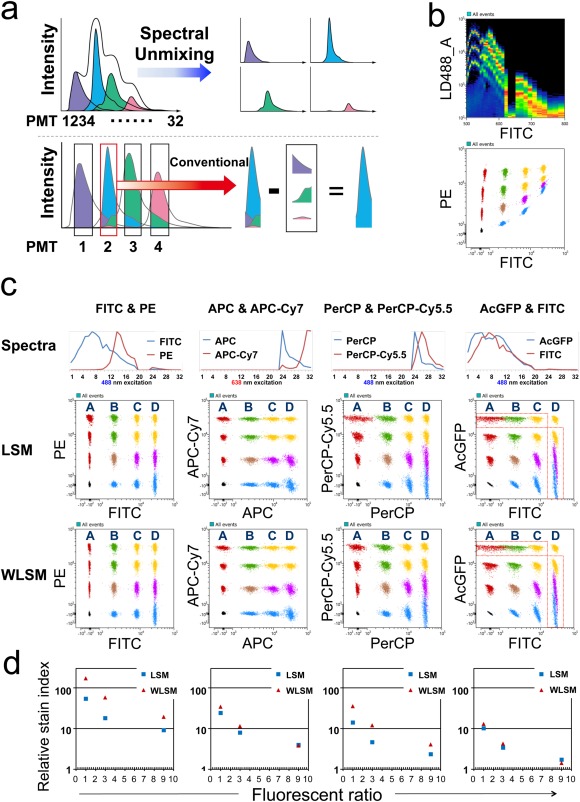
Principle of “Spectral Unmixing” and separation of fluoroprobes using *in silico* simulation. **a**) The spectral‐FCM unmixing algorithm utilizes all the emitted fluorescence as basic spectrum patterns to separate different spectrums (upper). On the other hand, conventional compensation utilizes only a narrow band of light to remove overlapping fluorescence emissions (lower). **b**) Spectrum chart by 488 nm laser excitation of simulated data using FITC and PE (upper). Fluorescent chart of simulated data before spectral unmixing (lower). To help identifying each population, some populations are painted with different colors. **c**) Spectra of combinations of FITC/PE, APC/APC‐Cy7, PerCP/PerCP‐Cy5.5, and AcGFP/FITC, respectively (upper). Normalized intensity spectra of each fluorochrome are shown as the ordinate. The intensity values of PMT channels from 20^th^ to 23^rd^ are assigned as zero because these simulation data were generated in dual laser mode which needs physical masking from 20^th^ to 23^rd^ in order to brock 638 nm laser. Fluorescent charts of FITC/PE, APC/APC‐Cy7, PerCP/PerCP‐Cy5.5, and AcGFP/FITC combinations after applying LSM and WLSM algorithm, respectively (lower). To help identifying each population in dot plots, some populations are painted with different colors. **d**) Comparison of relative stain index under changing fluorescent intensity ratio of two fluoroprobes; FITC/PE, APC/APC‐Cy7, PerCP/PerCP‐Cy5.5, and AcGFP/FITC after applying LSM and WLSM. Relative stain index is calculated using population “A and B,” “A and C,” and “A and D,” respectively. Fluorescent ratio of second fluoroprobe divided by first fluoroprobe as the abscissa and relative stain index as the ordinate are indicated.

The spectral‐FCM automatically analyzes acquired full spectrum data with unique algorithms based on the Least Squares Method (LSM) which enables separation of overlapping fluorescent spectra (Fig. 2a). The basis of our algorithm is as follows. Each spectrum derived from single stained samples and an unstained sample is recognized as the basic reference spectra. Then, multistained samples are mathematically fitted and unmixed using the component of each single stained and unstained reference spectra. Mathematically estimated coefficient values reflect the degree to which the multi stained sample consists of each spectrum. Then, we can simultaneously estimate the fluorescence intensity of each fluoroprobe in every cell without any complicated conventional compensation.

Let ***m*** *_i_* denote a base spectra of *K* channel, ***y*** *=(y_1_,…,y_K_)^T^* an observed spectrum of *K* channel where *^T^* denotes the transpose, $\omega_{i}$ a multiplier factor, *e* a noise, *M* the number of fluoroprobes, and then event data is represented as equation.

$$y=\sum_{i=1}^{M}\omega_{i}m_{i}+e$$

The base spectrum ***m*** *_i_* is identical to a normalized spectrum in some way by event data when one fluorochrome or FP passes through alone. Practically, the fluorescence spectra are usually calculated from an average of multiple event data of single stained sample using statistical processing. On the other hand, the multiplier factor $\omega_{i}$ is mathematically estimated, if an event data ***y*** and a base spectrum ***m*** *_i_* is given. The multiplier factor $\omega_{i}$ is called as the fluorescent intensity and is the most important number in FCM, as users often make a judgment about a measured sample by taking a look on the distribution of the $\omega_{i}$. LSM is a common way to determine $\omega_{i}$ and it can be obtained by the Eq. (33):

$$\hat{\omega }={argmin}_{\omega }{\left\|y-M\omega \right\|}^{2}$$

that is

$$\hat{\omega }={\left(M^{T}M\right)}^{-1}My$$

This equation could be also expressed as

$${\left\|y-M\omega \right\|}^{2}=\sum_{k=1}^{K}{\left(y_{k}-\sum_{i=1}^{M}\omega_{i}m_{ik}\right)}^{2}$$

Therefore, LSM simply estimates the amount of all fluoroprobes by minimizing residual signal and calculates only fluorescent intensities based on an assumption that the reference spectra impose equal weight for each detection channel.

However, it is well known that FCM data has heteroskedasticity which is based on larger noise in bright channels and smaller noise in dim channels. In order to impose proper weight for each detection channel, we developed a Weighted Least Square Method (WLSM) where $\lambda_{k}$ is weight for channel *K*:

$$\hat{\omega }={argmin}_{\omega }\sum_{k=1}^{K}\lambda_{k}{\left(y_{k}-\sum_{i=1}^{M}\omega_{i}m_{ik}\right)}^{2}$$

that is

$$\hat{\omega }={\left(M^{T}diag\left(\lambda \right)M\right)}^{-1}M^{T}diag\left(\lambda \right)y$$

where diag( λ) is diagonal matrix which has $\lambda ={\left(\lambda_{1},\ldots ,\lambda_{K}\right)}^{T}$ in a diagonal component. We also put in an offset in order not to make $\lambda_{k}$ too big or negative value when fluorescent signals are dim.

$$\lambda_{k}=\frac{1}{offset+max\left(y_{k},\;0\right)}$$

### *In Silico* Simulation

*In silico* simulation data sets were generated from synthesizing unstained sample data and single stained samples data. Unstained and single stained data were acquired by using fluorescent microbeads (BD FACS 7‐Color Setup Beads, BD Biosciences, CA) or green fluorescent protein (GFP) conjugated beads (AcGFP 34, 35 Flow Cytometer Calibration Beads, Clontech Laboratories Inc., CA). Synthesizing equation is below and we define *S*[ k] as spectra of events with *m* and *n* as the intensity ratio.

$$S_{result}\left[k\right]=m\times \left(S_{fluoroprobe1}\left[k\right]-S_{unstain}\left[k\right]\right)+n\times (S_{fluoroprobe2}\left[k\right]-S_{unstain}\left[k\right])+S_{unstain}[k]$$

It is important to note these simulation data sets are based on real acquired data, and include various noise factors and deviations related to both instrumental and sample conditions, such as laser RMS, noise of analog/digital conversion, digital filter, PMT voltage, flow rate, size, and so on.

We simulated four different combinations: fluorescein isothiocyanate (FITC)/R‐phycoerythrin (PE), allophycocyanin (APC)/APC‐Indotricarbocyanine (Cy)7, PerCP/PerCP‐Cy5.5, and AcGFP/FITC. These data were analyzed using two different algorithms (LSM and WLSM) and subpopulation signal separations were indicated by a relative Stain index. The relative Stain index is a normalized functional measure to quantify population resolution defined as below 36, 37. Higher relative stain index value means sharper resolution.

$$Stain\;index=\frac{D}{W}$$

$$\left(\begin{array}{l} D;\;\text{the}\;\text{difference}\;\text{between}\;\text{the}\;\text{mean}\;\text{fluorescence}\;\text{intensity}\;(\text{MFI})\;\text{of}\;\text{positive}\;\text{and}\;\text{negative}\;\text{population}\\ W;\;2\;\text{SD}\;\text{of}\;\text{the}\;\text{MFI}\;\text{observed}\;\text{for}\;\text{negative}\;\text{population} \end{array}\right)$$

### Mice and Cell Lines

Kaede transgenic mice have been reported previously 18. Establishment of ROSA‐CAG‐loxp‐stop‐loxp‐KikGR KI mice has also been described previously 19. KikGR mice were generated by mating ROSA‐CAG‐loxp‐stop‐loxp‐KikGR KI mice with CAG‐Cre mice 38. SCAT3.1 mice were generated by mating ROSA‐CAG‐loxp‐stop‐loxp‐SCAT3.1 knock‐in mice 39 with CAG‐Cre mice 38. FucciG_1_‐#639 and FucciS/G_2_/M‐#474 mice were established and reported previously 40. EGFP‐transgenic mice 41 were kind gift from M. Okabe (Osaka University, Japan). Langerin‐GFP mice 42 were kind gift from B. Malissen (INSERM‐CNRS‐Université de la Méditerranée Parc Scientifique et Technologique de Luminy, France). C57BL/6 mice were obtained from CREA Japan. The mice were maintained under specific pathogen‐free conditions, and the experimental procedures and housing conditions were approved by Kyoto University school of medicine, and all animals were cared for and treated humanely in accordance with the Institutional Guidelines for Experiments using Animals. The human cervical carcinoma cell line HeLa (American Type Culture Collection) was transfected with a CMV‐hKikGR‐cDNA (kindly provided by Dr. Atsushi Miyawaki) plasmid by FuGENE6 (Roche Diagnostics, Tokyo, Japan) and KikGR‐expressing HeLa cell line was established.

### Photoconversion

Kaede or KikGR expressing cells were exposed to violet light (95 mW/cm^2^ with a 436 nm bandpass filter with Spot UV curing equipment: SP‐9 Spot cure; USHIO Inc., Tokyo, Japan). Photoconversion of inguinal lymph node is previously described 18, 21, 43. Briefly KikGR mice were anesthetized and abdominal skin was cut at the midline to visualize the inguinal lymph node. After the surrounding tissue was covered with aluminum foil the lymph node was exposed to violet light through a hole in the foil with a continuous warmed PBS rinse. After photoconversion, the wound was closed with a suture.

### Flow Cytometry Analysis

**Figure 3:**
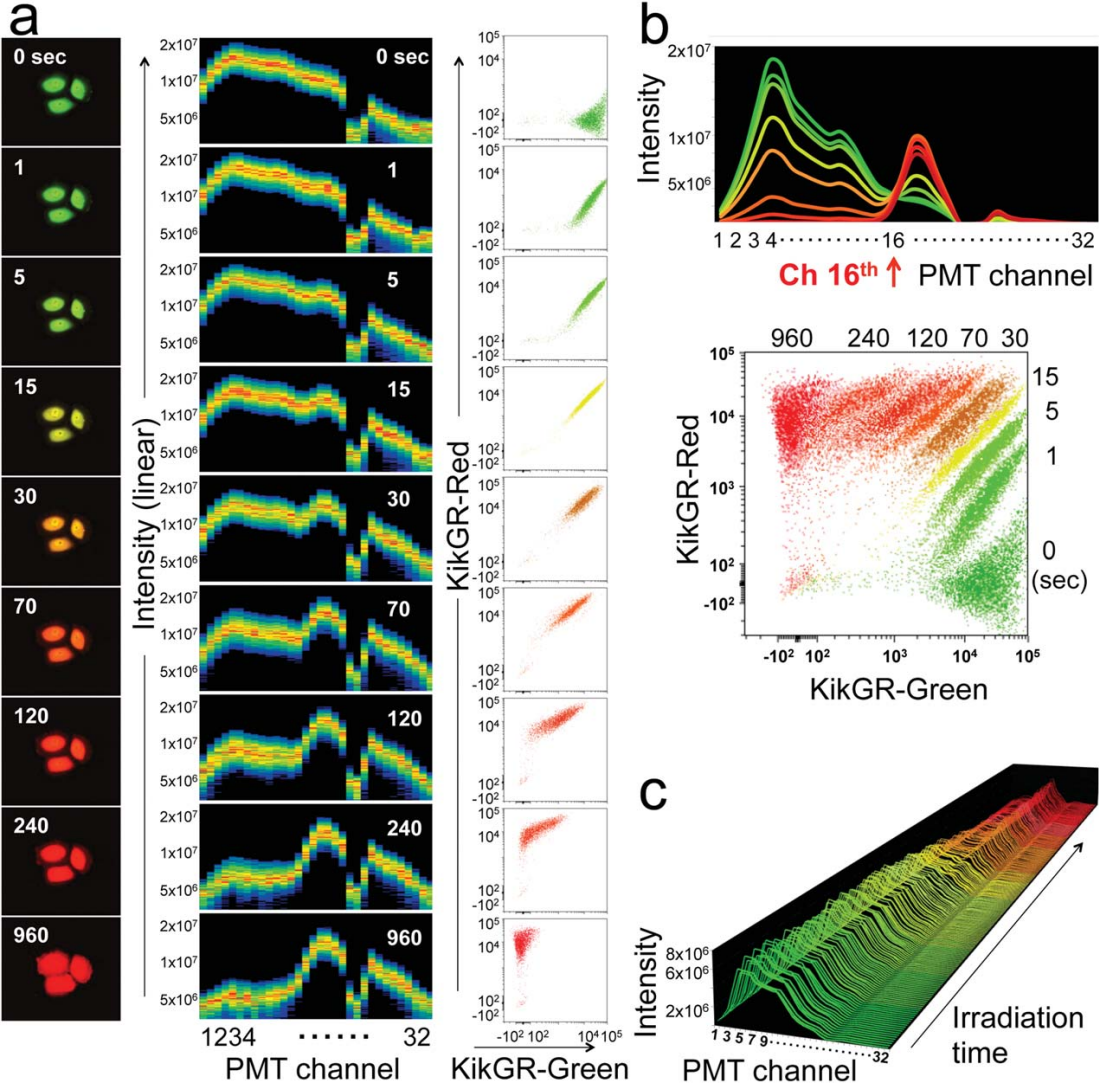
Detection of spectral changes of Kaede and KikGR expressing cells during photoconversion. **a**) Microscopic images after violet light irradiation for 0, 1, 5, 15, 30, 70 120, 240, or 960 sec (left). Spectral shapes with channels of PMT as the abscissa and fluorescent intensities as the ordinate are indicated (center). KikGR‐Green and KikGR‐Red intensities after Spectral Unmixing are shown in dot‐plots (right). Data are representative of two individual experiments. **b**) Spectral shapes and dot‐plots data unmixed with WLSM of Figure 3a are merged and shown, respectively. **c**) Irradiation time dependent spectral changes of splenocytes from Kaede‐transgenic mice during photoconversion. Splenocytes from Kaede‐transgenic mice in sample tube was placed in the spectral‐FCM and exposure to violet light during acquiring data. Data are representative of two individual experiments.

**Figure 4:**
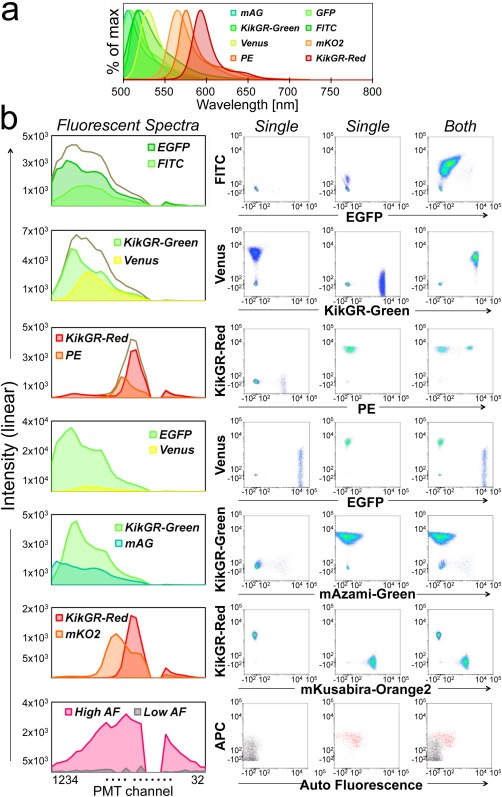
Separation of adjacent FP, fluorochrome, and autofluorescence. **a**) Spectra of FPs and fluorochromes. Normalized intensity spectrum of indicated FPs and fluorochromes are shown as the ordinate. mAzami‐Green and mKusabira‐Orange2 were indicated as mAG and mKO2, respectively. **b**) Separation results of spectrally‐adjacent FPs and fluorochromes. Fluorescent spectra charts show observed fluorescent intensities as the ordinate. Gray lines show the total fluorescence spectrum before unmixing. Separations of spectrally‐adjacent fluoroprobes with WLSM are shown in dot‐plots. In the panel of High/Low AF, fluorescence signal from APC‐conjugated anti‐mouse F4/80 mAb was applied as the ordinate because F4/80 is one of the major biomarkers of macrophage which has high autofluorescence (Supporting Information Fig. 4). Details of cells and fluorochrome‐conjugated antibodies used in this experiment are summarized in Supporting Information Table 2. Data are at least representative of two individual experiments.

**Figure 5:**
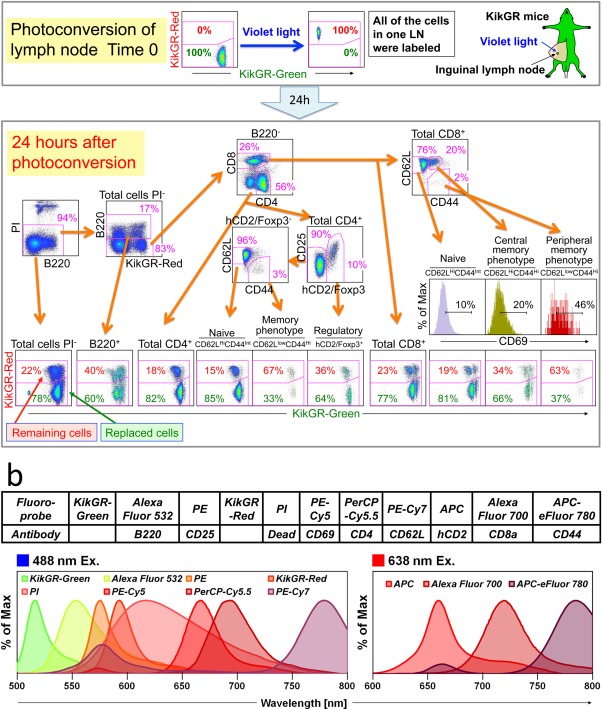
11‐Color analysis using KikGR mice. **a**) 11‐color analysis including two FPs and nine fluorochromes. The inguinal lymph node was exposed to violet light. Twenty‐four hours after photoconversion, cells from photoconverted inguinal lymph node were stained with fluorochrome‐conjugated antibodies and PI as described in **b**. Acquired data sets were unmixed with WLSM and are shown in dot‐plots. PI‐positive dead cells were gated out and B cells were gated as B220^+^ cells. B220^‐^ cells were gated into CD4^+^ and CD8^+^ cells. CD4^+^ cells were subdivided into hCD2/Foxp3^+^ regulatory T cells and hCD2/Foxp3^‐^ non‐regulatory T cells. hCD2/Foxp3^‐^ non‐regulatory T cells were further gated into naive (CD62L^High^CD44^Low^) and memory phenotype (CD62L^Low^CD44^High^) cells. On the other hand, CD8^+^ T cells were subdivided into naive, central memory (CD62L^High^CD44^High^), and peripheral memory (CD62L^Low^CD44^High^) phenotype cells. CD69 expressions in each CD8^+^ T‐cell subset are shown in histograms. Data are representative of two individual experiments. Intensities of stained fluorochromes (biexponential) for gating cell‐populations or fluorescent intensities of KikGR‐Green (biexponential) and KikGR‐Red (biexponential) in each population are shown in dot‐plots. Fluorescent intensities of CD69 (biexponential) as the abscissa are indicated in histograms. b) FPs and stained fluorochrome‐conjugated antibodies and PI and their spectrum. Normalized intensity spectrums of indicated FPs and fluorochromes are shown as the ordinate.

Antibodies used in this study were purchased from BioLegend, eBioScience, or BD Pharmingen, CA. Cells were washed with a staining buffer consisting of 2% fetal bovine serum and 0.02% sodium azide in Dulbecco's PBS treated with 2.4G2 hybridoma culture supernatant to block Fc biding, then stained with fluorochrome‐conjugated antibodies for 15 min on ice. After washing twice with staining buffer, propidium iodide (PI) was added and analyzed by the spectral‐FCM. All data was taken at the mid level flow rate (5 m/s). Flow cytometry data were analyzed with LSM and WLSM to compare results from beads (Figs. 2c and 2d). Data from cells were analyzed with WLSM in response to the comparison (Figs. 3, 4, 5).

### Microscopic Analysis

Cells were placed on a poly‐l‐lysine coated glass‐bottomed dish in a heated chamber set at 37 °C. KikGR‐Green was excited with 488 nm laser light and emission signal was obtained through a 525/50 band‐pass filter. KikGR‐Red was excited with 561 nm laser light and emission signal was obtained through a 575/25 band‐pass filter. Confocal images were acquired on an A1R MP (Nikon, Tokyo, Japan).

## Results

### Performances of the Spectral‐FCM

Flow cell chip alignment was automatically performed by analyzing fluorescent microbeads, Ultra Rainbow Fluorescent Particles (Spherotech, IL). Figure 1d shows spectral charts acquired by the detection of Ultra Rainbow Calibration Particles, eight peaks (Spherotech, IL). Molecules of equivalent soluble fluorochrome (MESF) were calculated in several regions to determine overall instrument sensitivity with a virtual filter calculation (FITC: 5–10 ch, 515.2–546.0 nm, PE: 14–18 ch, 565.9–607.4 nm, and APC: 24–25 ch, 662.4–688.5 nm). MESF‐FITC, MESF‐PE, and MESF‐APC were 100.3, 54.0, and 40.0, respectively (MESF‐PE, Fig. 1e). In addition, the linearity of signal for each calculated signal was good with *R* ^2^ values over 0.999. These values show the spectral‐FCM has as good sensitivity as a high end model conventional filter based FCM. By varying *m* and *n* values as well as source single stained samples, we generated varieties of simulated data. Figure 2b top shows a spectral chart of the combination of FITC and PE with 4 × 4 intensity levels. Figure 2b bottom shows raw data before applying an unmixing calculation. We also simulated 4 × 4 levels data for APC/APC‐Cy7, PerCP/PerCP‐Cy5.5, and AcGFP/FITC combinations. Figure 2c shows fluorescent charts of these data after unmixing algorithms were applied. Both algorithms could properly separate ordinal combinations such as FITC/PE and APC/APC‐Cy7. However, WLSM always produces better fitting than LSM because WLSM puts a higher weight on the detection channel with higher precision improving the quality of resolved populations. The separations with the higher relative stain index were obtained by applying WLSM in all cases (Fig. 2d). Thus, WLSM has been shown to be a more powerful and robust algorithm than LSM for these spectral flow data sets. Therefore we applied the WLSM algorithm to all experiments described in this article. We also found from the simulation that the increased fluorescent intensity ratio in spectrally‐adjacent and highly overlapping combinations, such as PerCP/PerCP‐Cy5.5 and AcGFP/FITC, makes accurate separation a little bit difficult because some populations were slightly spreading and resulted in lower relative stain index values than others (Figs. 2c and 2d).

To be clear, this is not traditional “Fluorescence Compensation,” as the applied technique is “Spectral Unmixing.” It is noteworthy that spectral‐FCM displays data plots, in real time, of not only spectral data, but also the fluorescent intensity of each fluorochrome after the spectral unmixing during data acquisition of cell samples. Therefore, it provides users with rapid, simple, and reliable analysis with a high degree of accuracy.

### Detection of Continuous Spectral Change of Photoconvertible FPs During Photoconversion

Irradiation of Kaede and KikGR with violet light induces an irreversible structural change of their chromophore 44, because of their one‐to‐one molecular reaction of chromophore change and continuous spectral changes from green to red in an exposure time‐dependent manner. Kaede and KikGR FPs are useful tools for evaluating software and hardware performance of the spectral‐FCM. We irradiated HeLa cells expressing KikGR (KikGR‐HeLa) or splenocytes of Kaede‐transgenic mice with violet light and analyzed KikGR and Kaede spectra during photoconversion. We detected increases of KikGR‐Red and Kaede‐Red signals together with the concomitant decrease of KikGR‐Green and Kaede‐Green spectra in a time dependent manner, respectively (Figs. 3a and 3c). The spectral changes of KikGR during photoconversion demonstrated an equivalent spectral shape obtained by the spectral‐FCM and the images obtained by microscopy (Fig. 3a). After 960 sec of irradiation, KikGR‐Green signal completely disappeared. Merged spectra charts and dot‐plots of KikGR‐Green and KikGR‐Red signals during photoconversion of KikGR‐HeLa indicated that the spectral‐FCM detected these slight spectra changes and then clearly distinguished each population from 0 to 960 sec of irradiation (Fig. 3b). In addition, because of their one‐to‐one molecular reaction of chromophore change, it was theoretically predicted and observed that all spectra from 0 to 960 sec crossed at same position “16th channel of the PMT” (Fig. 3b, red arrow). Furthermore, each population in the green versus red dot‐plot which has a linear‐linear axis showed rotational changes of the increasing KikGR‐Red signal together with the concomitant decreasing of KikGR‐Green signal (Supporting Information Fig. 1) 45, 46. Taken together, these results demonstrated the ability for high‐spectral resolution and reliability.

### Spectral Unmixing

By using immune cells from gene modified FP expressing mice and fluorochrome‐conjugated antibodies, we demonstrated the potential of the spectral‐FCM for separating the signal from FPs and fluorochromes despite highly overlapping fluorescent spectra that are hard to separate using conventional FCMs (Fig. 4a). Details of cells and fluorochrome‐conjugated antibodies used in this experiment were summarized in Supporting Information Table 2. In the case of EGFP (Em 507 nm) and FITC (Em 519 nm), although FITC signal slightly spilled over to EGFP signal, FITC^+^EGFP^‐^ population, and FITC^+^EGFP^+^ population were clearly distinguished even though there is only 8 nm difference between their emission peaks (Fig. 4b). In the combination of KikGR‐Green (Em 517 nm)/EYFP‐variant Venus (Em 528 nm) 47, we successfully separated KikGR‐Green^–^Venus^+^, KikGR‐Green^+^Venus^–^, and KikGR‐Green^+^Venus^+^ populations from single or double transgenic mice with slight spillover into the other parameter. We could detect expression variance of Venus among cell subsets, which indicated the potency of the spectral‐FCM for detecting small difference of signals under such a severe separating condition (Supporting Information Fig. 2). In the combination of PE (Em 578 nm)/KikGR‐Red (Em 591 nm), single positive and double positive populations were clearly separated without spillover. Although a large spillover of EGFP signal to Venus was observed in the combination of EGFP/Venus, still we had enough space to distinguish between double positive and single positives. Fucci protein is an effective probe for cell cycle phases, labelling individual G_1_ phase nuclei red (mKusabira‐Orange2, Em 565 nm) and those in S/G_2_/M phases green (mAzami‐Green, Em 505 nm) 48. Again, both KikGR‐Green/mAzami‐Green and KikGR‐Red/mKusabira‐Orange2 were effectively separated. Moreover, four FPs; KikGR‐Green, KikGR‐Red, mAzami‐Green, and mKusabira‐Orange2 were separated successfully from each other (data not shown). Success of simultaneous separation of these four FPs may have a potential application as a tool for revealing both spatiotemporal movement and cell cycle division of immune cells in the entire body.

Inherent cellular autofluorescence present in samples is a significant problem in many applications. It is well known that macrophages, monocytes, granulocytes and dying cells have high levels of cellular autofluorescence. We measured splenocytes from wild type mouse and found that high autofluorescence cells were observed only in a part of the F4/80^+^/CD11b^−^ MHC ClassII^high^ population, whereas, low cellular autofluorescence was observed in every population (Supporting Information Fig. 3). High cellular autofluorescence has broad curve spectrum with peak in around 580 nm and were clearly separated from low cellular autofluorescence cells with featureless spectral shape (Fig. 4b, bottom). These results indicated that the spectral‐FCM can distinguish cellular autofluorescence against each fluorochrome signal. Overall, the spectral‐FCM has successfully unmixed mutual overlaps of signals from every adjacent FPs, fluorochromes, and cellular autofluorescence even in heterogeneous population with heterogeneous fluorescent intensities.

### Simultaneous Detection of Cellular Movement in Multicellular Lineages by 11‐Color Analysis in KikGR Mice

Recently we have established KikGR mice 19 for analyzing spatiotemporal regulation of immune cells in addition to Kaede‐transgenic mice 18. For further detailed investigation of the dynamics of systemic immune cells, multiparameter analysis in the presence of KikGR‐Green and KikGR‐Red using FCM is required. We carried out 11‐color analysis using KikGR mice. When inguinal lymph node (LN) of KikGR mice was exposed to violet light for photoconversion of KikGR, all of the cells in inguinal LN were subsequently labeled with KikGR‐Red signal (Fig. 5a). Twenty‐four hours after photoconversion, we monitored cell migration from photoconverted LN to other anatomical organs (data not shown) and replacement of cells in photoconverted LN (Fig. 5a). Cells from inguinal LN 24 hours after photoconversion were stained with fluorochrome‐conjugated antibodies as shown in Figure 5b and analyzed. We have successfully unmixed heterogeneous fluorescence of individual cells into B cells and T cell subsets without complicated compensation. The KikGR‐Green^+^/KikGR‐Red^+^ population is remaining cells and KikGR‐Green^+^ population is cells replaced during 24 hours. The ratio between these populations indicated replacement rate in each population. CD69 expression on T cells, which gives us the information of interaction of T cells with endogenous antigen(s) 21, was also detected simultaneously. Frequency of CD69 expression was negatively correlated with replacement rate of T cells, thus, CD69 low naive T cell subset was replaced in LN faster than CD69 high memory T‐cell subsets (Tomura et al. 18, 21 and Fig. 5a). Thus, KikGR mice with the spectral technology allows us to analyze cellular dynamics and multiple functions in many subpopulations simultaneously controlling for changes in background and autofluorescence. Spectral‐FCM is a powerful tool to investigate immune system dynamics based on spatiotemporal regulation of immune cell subpopulations in the entire body.

It is noteworthy that every KikGR‐Green/KikGR‐Red double positive and KikGR‐Green single positive population in dot‐plots obtained from each subset were in the same relative position. These data indicated that the spectral‐FCM has the potent ability to perform reliable unmixing of multiple labeled, overlapping fluorochromes and FPs simultaneously.

## Discussion

Our novel cytometer based on spectral technology for multidimensional analysis is designed to overcome the limitations which conventional FCM faces with improvements to both hardware and software. In this study, we demonstrated that the unique prism optics and unmixing algorithm based on LSM enabled us to achieve high spectral resolution, reliable recognition of slight changes of FPs during photoconversion (Fig. 3) and unmixing of multiply labeled and overlapping fluoroprobes (Figs. 4 and 5). Key parameters for spectral unmixing are shapes of spectra, spectral overlap, and ratios of fluorescence. It is easy to unmix spectra when fluoroprobes have different shapes and small overlaps like combinations of FITC/PE and APC/APC‐Cy7 (Fig. 2c). On the other hand, in cases where fluoroprobes are spectrally adjacent and have large spectral overlap (like a combination of AcGFP/FITC), spectral unmixing becomes more difficult, especially with a large ratio of fluorescence (Fig. 2c, red dash line). Although, Green FPs, such as EGFP, KikGR‐Green, and mAzami‐Green have been developed to fit into the green channel of a fluorescent microscope and conventional FCM, their spectral peaks and shapes of each fluoroprobe are slightly different. We hypothesized that an unmixing algorithm could recognize each component and them unmix, if we could put a high enough resolution of wavelength profile in the blue‐green region of the PMT (Fig. 1b). As shown in Figure 4, a variety of combinations of adjacent fluoroprobes which emitted in blue‐green region were clearly unmixed. Moreover, although the spectra of EGFP and KikGR‐Green were almost the same, a spectral unmixing of KikGR‐Green and FITC was more difficult than that of EGFP and FITC (data not shown). It suggested that spectral unmixing algorithms recognized such a slight difference of spectra between EGFP and KikGR‐Green. This high spectral resolution of the spectral‐FCM was also indicated by the detection of the continuous spectral change of photoconvertible FPs during photoconversion (Fig. 3). Theoretically, WLSM is more powerful than LSM especially in the separation of dim samples, especially when compared with the separation result of FITC/PE and that of PerCP/PerCP‐Cy5.5 (Figs. 2c and 2d). Since the AcGFP spectrum almost fully covers the FITC spectrum, the separation of these spectra is more difficult and gives a lower relative stain index than the other combinations. In adjacent spectra separation, the limitation of fluorescent ratio between two fluoroprobes for the separation is around one to five times from *in silico* simulation (Fig. 2c). High fluorescent intensity ratio between fluoroprobes makes it more difficult to distinguish signals and noise that are mainly derived from the detection system and analog‐to‐digital conversion. The noise fluctuates in the spectrum for actual event data and causes spillover after applying spectral unmixing. Our unmixing algorithm identifies the spectral shape of each fluoroprobe, thus if the intensity value of two fluoroprobes identification was equal, spillover ought to occur both‐ways equally. However, one‐way spillover occurs in some combinations such as APC and APC‐Cy7. APC has major red peak around 660 nm. On the other hand, APC‐Cy7 has minor peak of APC around 660 nm and major far‐red peak of Cy7. Thus, the APC‐Cy7 spectrum encompasses the APC spectrum. In the LSM based spectral unmixing algorithm, complementation of the encompassed two peak spectrum (APC‐Cy7) by one peak spectrum (APC) is relatively harder and the opposite way is easier. This results in causing one‐way spillover with populations in the combination of APC/APC‐Cy7 creating a horizontally long elliptical shape (Fig. 2c). On the other hand, vertically long elliptical shapes were observed in the combination of FITC/PE. This is because FITC encompasses PE spectrally.

We also confirmed that unmixed resolution had increased with the increasing amount of information in practice as well as in theory using beads and simulation data, in case of tandem fluorochromes such as PE‐Cy5 and PE‐Cy7 detection, which the spectral‐FCM detects both emitted fluorescence signals from 488 nm and 638 nm laser excitations (data not shown). Sharpness of spectrum increased (about 10% in stain index) by adding data from 638 nm laser excitations to data from 488 nm laser excitations. It suggested that dual detection of fluorescent signals from tandem fluorochromes by two lasers reinforces spectral unmixing in our system.

A spreading spillover with the increasing of fluorescent intensity can happen in practical (not ideal) conditions, and when combined with the difficulty of visual estimation of proper compensation due to artifacts from log‐transformation can lead to overcompensation by visual adjustment using conventional FCMs 49. On the other hand, LSM and WLSM unmix spectra mathematically and give unmixed data directly. This means that there is no need to manually estimate proper compensation like conventional FCM. Unmixed data from synthesizing EGFP and Venus spectra in different fluorescent intensities shows increased symmetrical spreads of unmixed data with increasing fluorescent intensities (Supporting Information Fig. 4). In addition, each single spectrum can be shown by gating. This means that each single dot can be checked whether it is reliable fluorescence data or just a spike of noise (Supporting Information Fig. 5). This feature is very useful especially in rare cell analysis to improve enumeration and overall confidence in specificity. Overall, the spectral‐FCM has high reliability without arbitrariness.

As shown in Figure 4b, we could detect cellular autofluorescence. Different cell populations had unique autofluorescence spectra. Thus, it can be possible to distinguish cells by their autofluorescence fingerprints without surface antibody or intracellular staining that might cause unintended pleiotropic cellular responses. In fact, it is already discussed that specific cells differentiated from stem cells can be identified by autofluorescence fingerprints for therapeutic value 50 and the spectral‐FCM achieves this approach.

The immune system is a highly complicated system distributed throughout the entire body. Many kinds of immune cells move between organs to receive appropriate signals for their survival and expression functions. Thus in order to understand the immune system it is critical to reveal the mutual spatiotemporal regulation among various immune cells. New methods to visualize multilinage immune cells by applying spectral technology with photoconvertible FP, KikGR mice, and multiple fluorochromes as shown in this study may give us a great advance to grasp whole images of immune cell dynamics in vivo **(**Fig. 5). Furthermore, the next full‐spectral‐FCM utilizing a 3‐laser system will broaden the combinational use of KikGR mice together with FPs such as ECFP/EYFP FRET for detection of cell signaling, which will make possible to reveal the correlation of cellular movement and signaling in the same cells. Moreover, combined with a cell sorter system, it will realize true multidimensional single cell mapping by connecting information of proteomics and genomics. Overall, the spectral flow cytometry presented here open the door to high throughput and multicolor analyses of spectrally‐adjacent FPs and fluorochromes. It is a potentially transformative technology that facilitates investigation in many research fields.

## Supporting information

Supplementary Information

Supplementary Information
