# Supplementary material for: Novel full‐spectral flow cytometry with multiple spectrally‐adjacent fluorescent proteins and fluorochromes and visualization of in vivo cellular movement
Source: Cytometry A. 2015 Jul 28;87(9):830–42. doi: 10.1002/cyto.a.22725 (PMC5132038; doi:10.1002/cyto.a.22725)
Supplement: Supplementary file 1 — Supplementary Information [file CYTO-87-830-s001.pdf]

Supplementary Table 1. The specifications of the spectral-FCM

Optics	Excitation	Wavelength	488 nm LD / 638 nm LD
		Power	40 mW (488 nm), 60 mW (638 nm)
		Beam spot	Separation
		Beam spot shape	Orthogonal flow direction: 40 µm (Top Hat)<br>Flow direction: 6 µm (Gaussian)
	Detection	Scattering	FSC: a-Si PD (0.5~40 µm particle size)
			SSC: a-Si PD
			Flow position of cell : quadrant a-Si PD
		Fluorescence	Independent voltage controllable 32ch PMT
		Wavelength range	500 – 800 nm
Fluidics	Velocity		Low (3 m/s), Mid (5 m/s), High (10 m/s)
	Sample flow rate		18 µl/min, 30 µl/min, 60 µl/min
	Event rate		10,000 event per second
	Flow cell chip	Size	25 mm*75 mm*2 mm
		Cross section of detection area	200 µm*200 µm
		Sample core diameter	10 µmϕ (nominal)
		Function	Exchangeable , automated chip alignment
Electronics	Data resolution		Height 20 bit, Area 32 bit<br>Sampling frequency: 50 MHz

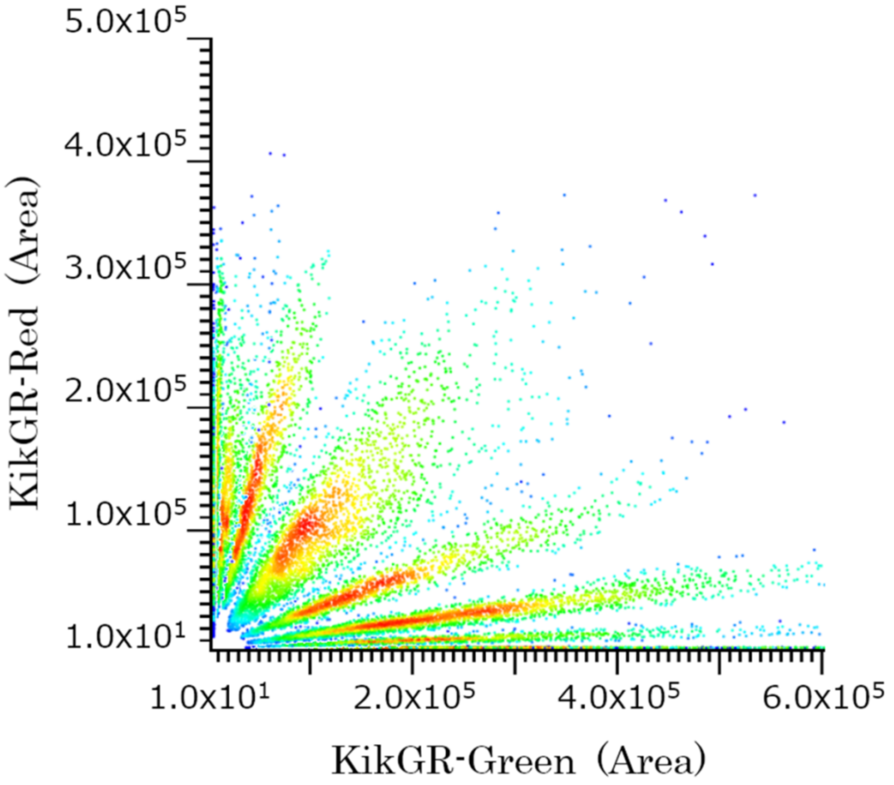

Supplementary Figure 1. Rotational changes of dot plots during photoconversion in linear-linear axis of KikGR-Green and KikGF-Red. Dot-plots data of **Fig. 3a** are merged and shown in linear-linear axis. Because the photoconversion of KikGR occurs as a one-to-one molecular reaction of chromophore change dot plots during photoconversion move around centered at the origin[9, 10].

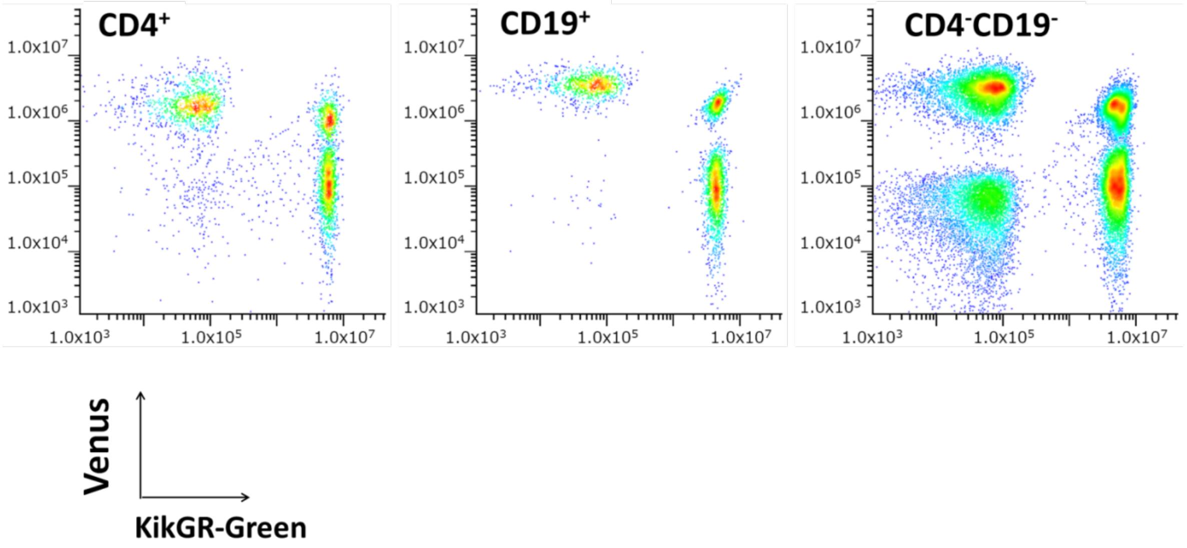

Supplementary Figure 2. Difference among population of separation for KikGR-Green/Venus. Splenocytes from KikGR mice, SCAT3.1 mice and KikGR/SCAT3.1 mice were stained with APC-conjugated anti-CD4 mAb and APC-Cy7-conjugated anti-CD19 mAb and analyzed by the spectral-FCM. These cells were divided into 3 populations, $CD4^{+}$, $CD19^{+}$, and $CD4^{-}CD19^{-}$ and intensities of KikGR-green and Venus are shown in dot-plot. In each population, KikGR-Green and Venus have different fluorescent intensities and variances. Data are representative of two individual experiments.

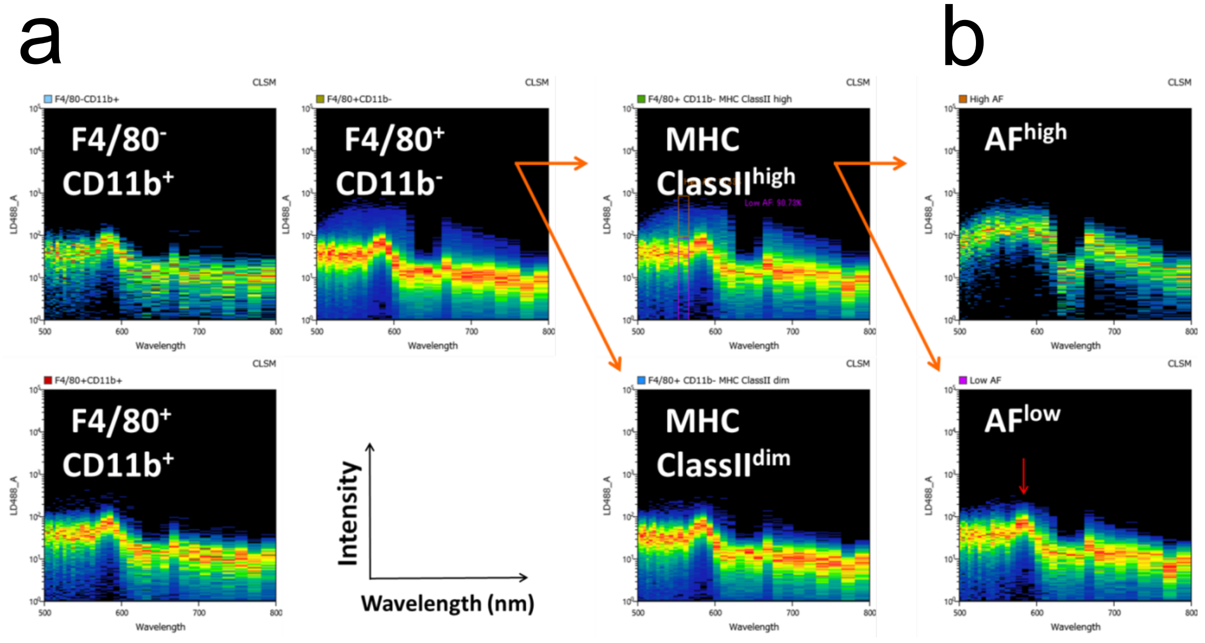

Supplementary Figure 3. Spectrum derived from cellular autofluorescence. Splenocytes from wild type mice were stained with APC-conjugated anti-F4/80 mAb, Alexa Fluor® 700-conjugated anti-MHC Class II mAb, and APC-Cy7-conjugated anti-CD11b mAb and analyzed by the spectral-FCM. Emission spectra with 488 nm laser excitation are shown. a) Spectrum of F4/80^{-}CD11b^{+}, F4/80^{+}CD11b^{+}, F4/80^{+}CD11b^{-}, F4/80^{+}CD11b^{-}Class II^{high} and F4/80^{+}CD11b^{-}Class II^{dim} are shown. Broad curve spectrum derived high cellular autofluorescence was observed in F4/80^{+}CD11b^{-}MHC Class II^{high} population. b) Comparison between high autofluorescence and low autofluorescence. Narrow peak observed around 580 nm (red arrow) is water Raman scatter, not cellular AF. Data are representative of three individual experiments.

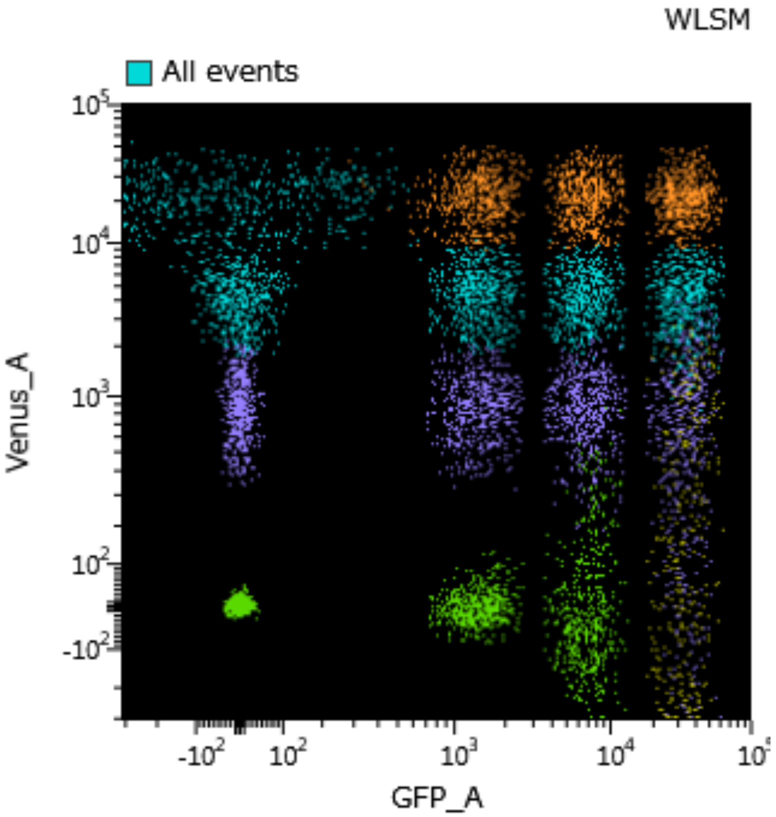

Supplementary Figure 4. Unmixed data of the simulated data from EGFP and Venus. Simulated data was generated from synthesizing unstained sample data, EGFP single stain data, and Venus single stain data which were used in **Fig. 4b** with the equation mentioned in **Methods: *In silico* simulation**. There are 4*4=16 populations in this dot plot. To help identifying each population, some populations are painted with different colors.

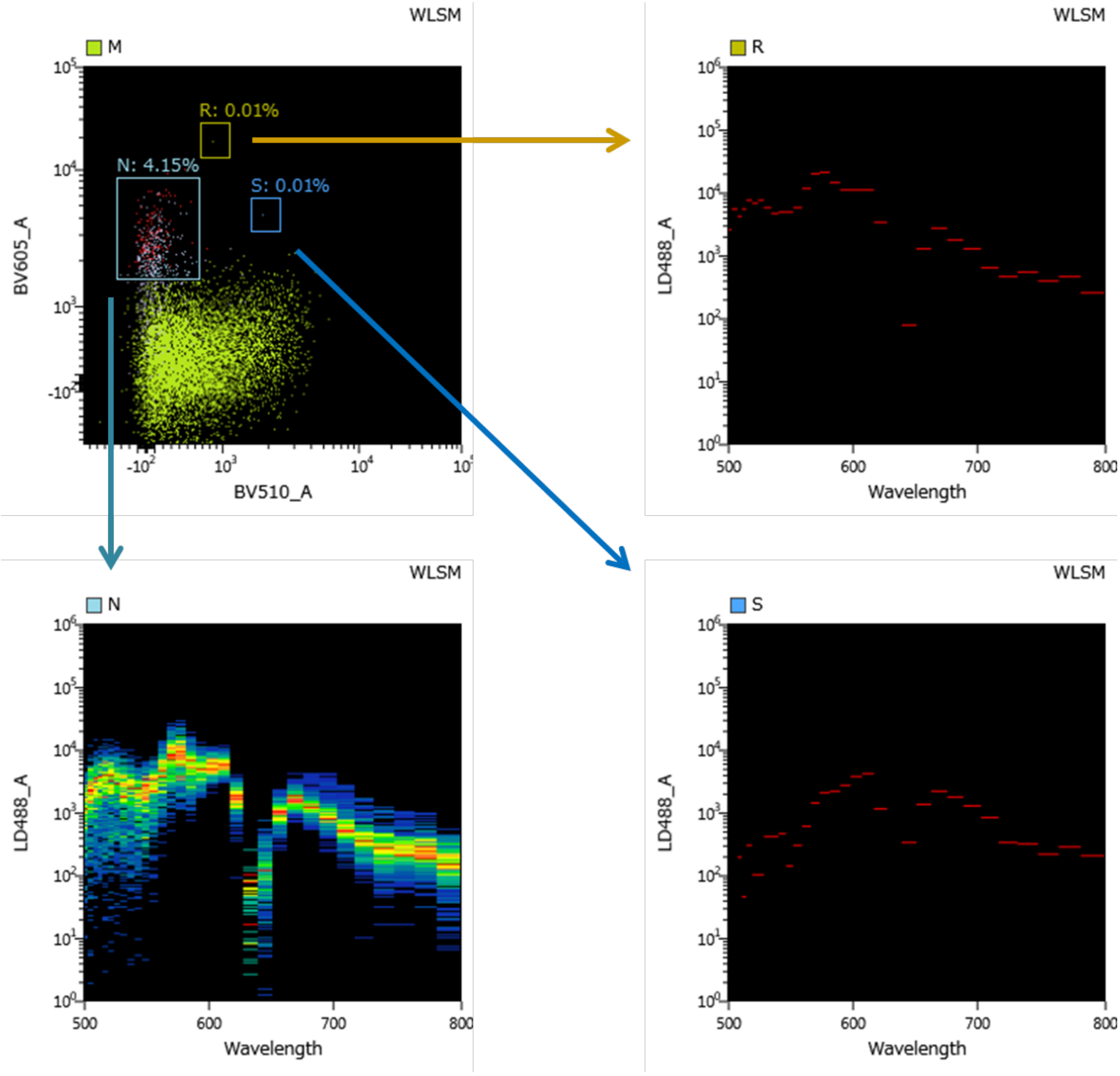

Supplementary Figure 5. Spectral shapes in different populations.
Gate N shows merged spectra of certain amount of cells. On the other hand, Gate R and S show only one spectrum observed from one cell, respectively.

Supplementary Table 2. Summary of cells and fluorochrome-conjugated antibodies in the experiment of separation of spectrally-adjacent fluoroprobes

Combinations		Fluorescents	Cells
EGFP / FITC	Single	Langerin-EGFP	CD11c$^+$ lymph node cells of Langerin-EGFP mice[1]
	Single	FITC-anti-CD8 mAb	Lymph node cells of wild-type mice
	Both	Langerin-EGFP / FITC-CD45	CD11c$^+$ lymph node cells of Langerin-EGFP mice[1]
KikGR-Green / Venus	Single	Venus	CD19$^+$ lymph node cells of KikGR/SCAT3.1 mice[3,4]
	Single	KikGR-Green[2]	CD19$^+$ lymph node cells of KikGR mice[3]
	Both	Venus / KikGR-Green[2]	CD19$^+$ lymph node cells of KikGR/SCAT3.1 mice[3,4]
KikGR-Red/PE	Single	KikGR-Red[5]	Lymph node cells of KikGR mice
	Single	PE-anti-CD8 mAb	Lymph node cells of wild-type mice
	Both	KikGR-Red / PE	Lymph node cells of KikGR mice
EGFP / Venus	Single	EGFP	Lymph node cells of EGFP-Tg mice
	Single	Venus	Lymph node cells of SCAT3.1 mice[4]
	Both	EGFP / Venus	Lymph node cells of EGFP-Tg mice<br>Lymph node cells of SCAT3.1 mice[4]
mAzami-Green / KikGR-Green	Single	mAzamiGreen	Lymph node cells of FucciS/G$_2$/M-#474 mice
	Single	KikGR-Green[2]	Lymph node cells of KikGR mice
	Both	mAzami-Green / KikGR-Green	Lymph node cells of FucciS/G$_2$/M-#474 mice<br>Lymph node cells of KikGR mice
mKusabira-Orange2 / KikGR-Red	Single	mKusabira-Orange2	Lymph node cells of FucciG$_1$-#639 mice
	Single	KikGR-Red[5]	Lymph node cells of KikGR mice
	Both	mKusabira-Orange2 / KikGR-Red[4]	Lymph node cells of FucciG$_1$-#639 mice<br>Lymph node cells of KikGR mice
Autofluorescence high / low	Single	Autofluorescence low	Spleen cells of wild-type mice
	Single	Autofluorescence high	Spleen cells of wild-type mice
	Both	Autofluorescence high / low	Spleen cells of wild-type mice

1. Cells were stained with APC-Cy7-conjugated anti-CD11c monoclonal antibody and gated CD11c$^+$ cells.
2. KikGR-Green: Cells were used without photoconversion.
3. SCAT3.1 molecules is fusion protein of ECFP and Venus binding with caspase-3 cleavable peptide. We confirmed fluorescent spectrum excited with 488 nm laser of SCAT3.1 is almost identical to that of Venus (data not shown) and used SCAT3.1 for fluorescent signal source of Venus.
4. Cells were stained with APC-conjugated anti-CD19 monoclonal antibody and gated CD19$^+$ cells.
5. KikGR-Red : Cells from KikGR mice were irradiated with violet light (436 nm, 100 mW/cm^2, 960 sec). After irradiation of this condition, green fluorescence disappeared as shown in Fig. 3a,b and photoconversion of KikGR completed.
