# Supplementary material for: Novel full‐spectral flow cytometry with multiple spectrally‐adjacent fluorescent proteins and fluorochromes and visualization of in vivo cellular movement
Source: Cytometry A. 2015 Jul 28;87(9):830–42. doi: 10.1002/cyto.a.22725 (PMC5132038; doi:10.1002/cyto.a.22725)
Supplement: Supplementary file 2 — Supplementary Information [file CYTO-87-830-s002.doc]

**Cytometry Part A**

**Author Checklist: MIFlowCyt-Compliant Items**

| **Requirement** | **Please Include Requested Information** |
| --- | --- |
| 1.1. Purpose | The purpose of this experiment was to show the potential of a novel spectral flow cytometry for the investigation of immune system using photoconvertible fluorescent proteins and multiple fluorochromes. |
| 1.2. Keywords | Spectral flow cytometry, Separation of spectrally-adjacent fluorescent probes, Photoconvertble fluorescent protein, Immune cell movement |
| 1.3. Experiment variables |  |
| 1.4. Organization name and address | Name:  Koji Futamura^1^, Masashi Sekino^2^, Akihiro Hata^3^, Ryoyo Ikebuchi^3^, Yasutaka Nakanishi^3^, Gyohei Egawa^4^, Kenji Kabashima^4^, Takeshi Watanabe^3^, Motohiro Furuki^1^, Michio Tomura^3^  Address:  ^1^FCM Business Department, Life Science Business Division, Medical Business Unit, Sony Corporation, 1-7-1 Konan, Minato-ku, Tokyo, 108-0075, JAPAN  ^2^Concept Development Department, Application Technology Development Division, System R&D Group, RDS Platform, Sony Corporation, 5-11-3 Kitashinagawa, Shinagawa-ku, Tokyo, 141-0001, JAPAN  ^3^Center for innovation in Immunoregulative Technology and Therapeutics, Kyoto University Graduate School of Medicine, Yoshida-Konoe, Kyoto 606-8501, JAPAN  ^4^Department of Dermatology, Kyoto University Graduate School of Medicine, 54 Shogoin-Kawara, Kyoto 606-8501, JAPAN |
| 1.5. Primary contact name and email address | Name:  Michio Tomura, Ph.D.  Email:  michio.tomura@gmail.com |
| 1.6. Date or time period of experiment | The experiments were performed between 2012 and 2013. |
| 1.7. Conclusions | New methods to visualize multi-linage immune cells by applying spectral technology with photoconvertible FP, KikGR mice and multiple fluorochromes as shown in this study may give us a great advance to grasp whole images of immune cell dynamics *in vivo*. |
| 1.8. Quality control measures | Optics alignment was performed automatically using Ultra Rainbow Fluorescent Particles (Spherotech, IL, USA). Sensitivities in FITC, PE, and APC regions were checked by Ultra Rainbow Calibration Particles, 8 peaks (Spherotech, IL, USA). |
| 2.1.1.1. (2.1.2.1., 2.1.3.1.) Sample description |  |
| 2.1.1.2. Biological sample source description | Kaede transgenic mice were reported previously(18). Establishment of ROSA-CAG-loxp-stop-loxp-KikGR KI mice is described previously(19). KikGR mice were generated by mating ROSA-CAG-loxp-stop-loxp-KikGR KI mice with CAG-Cre mice(38). SCAT3.1 mice were generated by mating ROSA-CAG-loxp-stop-loxp-SCAT3.1 knock-in mice(39) with CAG-Cre mice(38) and used for experiments. FucciG1-#639 and FucciS/G2/M-#474 mice were established and reported previously(40). EGFP-transgenic mice(41) were kindly provided by Dr. Masaru Okabe (Osaka University, Japan). Langerin-GFP mice(42) were kindly provided by Dr. Bernard Malissen (INSERM-CNRS-Université de la Méditerranée Parc Scientifique et Technologique de Luminy, France). C57BL/6 mice were obtained from CREA Japan. The human cervical carcinoma cell line HeLa (American Type Culture Collection) was transfected with CMV-hKikGR-cDNA (Kindly provided by Dr. Atsushi Miyawaki) plasmid by FuGENE6 (Roche Diagnostics, Tokyo, Japan) and KikGR-expressing HeLa cell line were established. |
| 2.1.1.3. Biological sample source organism description | Human/mouse |
| 2.1.2.2. Environmental sample location |  |
| 2.3. Sample treatment description |  |
| 2.4. Fluorescence reagent(s) description | Summarized in Figure 5b and Supplementary Table 2. |
| 3.1. Instrument manufacturer | Sony Corporation http://www.sonybiotechnology.com/index.php |
| 3.2. Instrument model | SP6800 |
| 3.3. Instrument configuration and settings | 488 and 638 nm lasers, 32 channel PMT  Details were summarized in Supplementary Table 1 |
| 4.1. List-mode data files | Sony raw data file can be obtained by contacting Dr. Michio Tomura. |
| 4.2. Compensation description | Spectral unmixing based on least square method was applied. |
| 4.3. Data transformation details |  |
| 4.4.1. Gate description | Doublet population was excluded by FSC-H/FSC-W and SSC-H/SSC-W. |
| 4.4.2. Gate statistics |  |
| 4.4.3. Gate boundaries |  |

**Notes**

Feel free to use more space than allocated.

You can embed graphics/figures in this document, if needed.

Please make sure to save the document in Microsoft Word version 2003 or older, before uploading to ScholarOne Manuscripts. When uploading this checklist to ScholarOne Manuscripts, please choose the “Supplementary Material for Review” category.

Please note that if your paper is accepted, the checklist will be published as an Online Supporting Information.

For any questions, please contact the Cytometry Part A editorial office at [Cytometrya@wiley.com](mailto:Cytometrya@wiley.com).
